# Supplementary material for: Disentangling metabolic functions of bacteria in the honey bee gut
Source: PLoS Biol. 2017 Dec 12;15(12):e2003467. doi: 10.1371/journal.pbio.2003467 (PMC5726620; doi:10.1371/journal.pbio.2003467)

uoro−11beta,16alpha,17alpha,21−tetrahydroxypregn−4-
# 778 395.187 microbial product

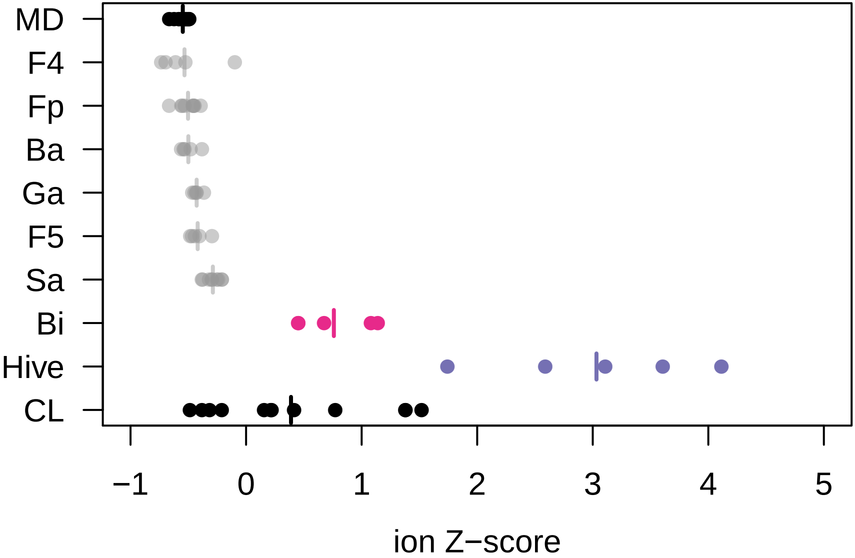

Supplement: S8 Data — (ZIP) [file pbio.2003467.s008.zip › Z-score_plots/778 microbial product 9alpha-Fluoro-11beta,16alpha,17alpha,21-tetrahydroxypregn-4-ene-3,20-dione.pdf]
